# A Rare Case of Cutaneous Sporotrichoid Aspergillus ustus Infection in a Transplant Patient

**DOI:** 10.7759/cureus.105618

**Published:** 2026-03-21

**Authors:** Catherine Seelig, Ted Rosen

**Affiliations:** 1 School of Medicine, Baylor College of Medicine, Houston, USA; 2 Dermatology, Baylor College of Medicine, Houston, USA

**Keywords:** aspergillus, aspergillus ustus, cutaneous aspergillosis, cutaneous mycoses, immunocompromised host, lymphangitis, lymphocutaneous, sporotrichoid pattern

## Abstract

*Aspergillus *spp. have long been recognized as human pathogens and are found ubiquitously. Cutaneous infections are an uncommon manifestation of aspergillosis and very rarely present with a sporotrichoid pattern of spread. We present a case of a 50-year-old immunocompromised man with a heart transplant who sustained trauma to his right ankle three weeks prior to presentation. The lesion was subsequently exposed to stagnant water, and he developed a draining nodule at the site of trauma. He soon developed ipsilateral, ascending nodules spreading in a sporotrichoid pattern. A combination of pathology and microbiology was utilized to diagnose the causative organism to be *Aspergillus ustus*, and fungal sensitivities were vital to provide appropriate treatment. This case is a rare manifestation of aspergillosis. There have only been four other reported cases of cutaneous aspergillosis presenting with a sporotrichoid pattern of spread, and none of these cases identified *Aspergillus ustus *as the causative organism. This case highlights the importance of utilizing a comprehensive diagnostic approach to diagnose atypical cutaneous infections, as well as the importance of fungal sensitivities to guide effective treatment.

## Introduction

*Aspergillus *spp. were one of the first mycoses recognized, with the first human case reported in 1847 [1]. There are approximately 465 recognized species found worldwide [2]. Of these, it is estimated that fewer than 40 cause human disease [3]. *Aspergillus fumigatus* and *Aspergillus flavus* are the two most common human pathogens. Other human pathogens include *Aspergillus niger *and *Aspergillus terreus*, which are generally seen in immunocompromised individuals, and, less commonly, *Aspergillus nidulans* [4]. *Aspergillus *infections occur predominantly in immunocompromised populations such as solid organ transplant recipients, patients with hematologic malignancies, and patients on prolonged immunosuppressive therapy [1].

Cutaneous manifestations of aspergillosis are rare. Primary cutaneous aspergillosis arises from direct inoculation to the skin through lacerations, burns, intravenous catheters, and other injuries to the skin. It commonly presents as a necrotic, hyperpigmented plaque or nodule with dark scaling. Secondary cutaneous aspergillosis is more common, with the primary infection occurring in the lungs. Disseminated infections can involve the central nervous system, kidneys, and heart [1].

Histologically, cutaneous aspergillosis typically presents as hyaline, septate hyphae with 45° dichotomous branching. The organism is best visualized using Gomori methenamine silver (GMS) or periodic-acid-Schiff (PAS) stains [1]. First-line therapy generally involves triazoles, with amphotericin B or echinocandins available as secondary treatment options [5].

A sporotrichoid pattern of spread is a very distinct clinical presentation caused by a limited number of organisms, most notably, *Sporothrix schenckii*. This cutaneous infection starts at the site of inoculation and spreads proximally along lymphatic channels, leading to nodular lymphangitis [6]. *Aspergillus *spp. are very rarely reported to be the cause of this clinical presentation.

This report presents the case of a patient with a rare manifestation of cutaneous sporotrichoid aspergillosis caused by *Aspergillus ustus*. This case underscores the critical importance of utilizing a combination of diagnostic techniques and antifungal sensitivities in a management strategy when faced with an uncommon cutaneous presentation.

## Case presentation

The patient was a 50-year-old immunocompromised Black man who presented for evaluation of tender, ascending nodules on his right lower extremity. His medical history included a heart transplant complicated by chronic heart failure, cardiomegaly, and severe hypertension; chronic kidney disease on hemodialysis; prior deep vein thrombosis with pulmonary embolism; cerebral vascular accident; gout; dyslipidemia; anemia of chronic disease; and sleep apnea. His immunosuppressive, anti-rejection medications included prednisone 2.5 mg daily, tacrolimus 2.0 mg daily, and mycophenolate mofetil 1500 mg twice daily.

**Figure 1:**
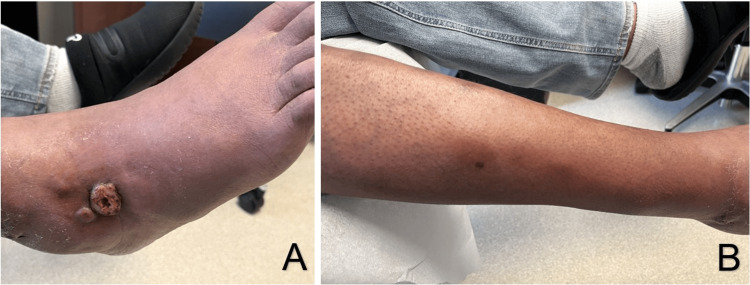
(A) Initial right lower extremity lesion. (B) Proximal, ascending lesions spread in a sporotrichoid pattern.

Three weeks prior to presentation, he hit his right ankle on a tub. The lesion was later exposed to stagnant water, and he developed a tender, draining nodule. Over the following days, additional ipsilateral nodules appeared in an ascending, sporotrichoid pattern of spread. On presentation to the hospital, the initial site of trauma revealed a 3×3 cm ulcerated, exophytic nodule with associated ascending, deep-seated nodules ranging in size from 3.4x3 cm to 4x3 cm (Figure 1A, 1B). The patient reported no fevers and felt “well” during this time. His vitals were stable with mild hypertension. Laboratory values were consistent with renal failure and mild anemia. Liver function test, electrolytes, and white blood cell count were unremarkable, as seen in Table 1. He was admitted to the hospital due to his many chronic medical issues.

**Table 1:** Vitals and laboratory values on admission

Laboratory Test	Value
Respiration	16 breaths per minute
Pulse	90 beats per minute
Temperature	97.4⁰F
Blood pressure	133/91 mmHg
Hemoglobin, blood	13.1 g/dL
Hematocrit	41.10%
Leukocyte count	5300 cells/mm^3^
Electrolytes	Unremarkable
Blood urea nitrogen	60 mg/dL
Creatinine	8.23 mg/dL
Glucose	120 mg/dL
Estimated glomerular filtration rate (EGFR)	<15 mL/minute/1.73 m^2^
Serum calcium	Unremarkable
Liver function test	Unremarkable

The initial differential diagnosis for a post-traumatic lesion with environmental exposure and sporotrichoid pattern of spread included several infections such as sporotrichosis, histoplasmosis, blastomycosis, cryptococcosis, *Mycobacterium marinum*, *Mycobacterium chelonae*, *Nocardia brasiliensis*, *Nocardia asteroids*, leishmaniasis, and cutaneous tuberculosis. The initial concern was primarily for sporotrichosis, considering the clinical morphology.

Punch biopsies of the initial lesion and a more proximal lesion were obtained and submitted for testing. Requested tests included hematoxylin and eosin (H&E) stain, GMS stain, PAS stain, acid-fast bacilli (AFB) stain, Fite stain, Gram stain, immunohistochemistry (IHC), fungal cultures, and mycobacterial cultures. A fungal DNA polymerase chain reaction (PCR) test was sent to the University of Washington. While the tests were pending, the patient was treated with thermotherapy. A Sunbeam-brand heating pad (Boca Raton, FL) was used on high (roughly 140°F) for 15 minutes, three times a day.

**Figure 2:**
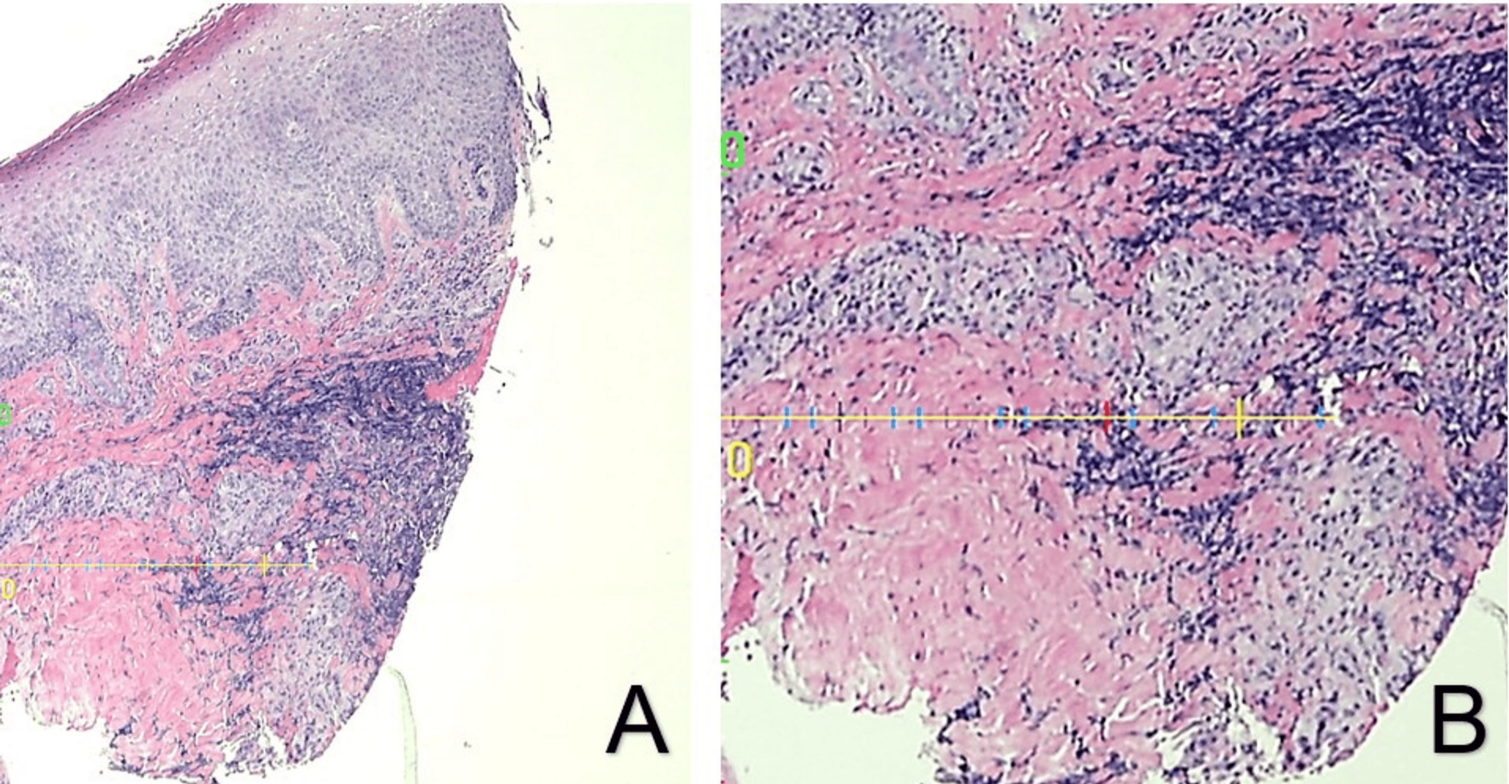
(A) Hematoxylin-eosin, magnification ×10. (B) Same slide, higher magnification.

**Figure 3:**
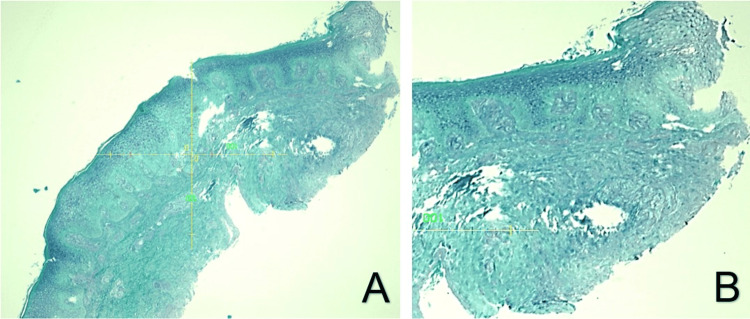
(A) Gomori methenamine silver (GMS) stain, magnified ×10, failing to identify fungi. (B) Same slide, higher magnification.

The initial pathology report stated, “Acanthosis, parakeratosis, dermal fibrosis with a mixed perivascular inflammatory infiltrate. Clinical correlation is indicated.” An addendum later stated, “Scant, poorly formed granulomas and dermal abscess formation. Correlation with culture and other microbiological studies is recommended.” Histology is depicted in Figure 2A, 2B. PCR testing returned negative for sporotrichosis, histoplasmosis, blastomycosis, *Coccidioides*, *Cryptococcus*, and *Mycobacterium marinum*. Immunohistochemistry returned negative for sporotrichosis and mycobacteria. GMS stain failed to identify a fungal agent and is depicted in Figure 3A, 3B. Fungal cultures from both lesions revealed *Aspergillus ustus*, identified by colonial morphology and confirmed by DNA sequencing of the internal transcribed spacer region and calmodulin gene. Further testing with a Fungitell test was negative, greatly reducing the likelihood of fungemia. Antifungal sensitivities came back, as seen in Table 2.

**Table 2:** Antifungal sensitivities MIC = minimum inhibitory concentration.

Agent	Case MIC (µg/mL)	Reference MIC90 (µg/mL) [7]
Amphotericin B	4	0.5
Micafungin	0.03	-
Fluconazole	>64	-
Itraconazole	>64	-
Posaconazole	2	8
Voriconazole	4	2
Isavuconazole	Requested, but not reported	2-4

**Figure 4:**
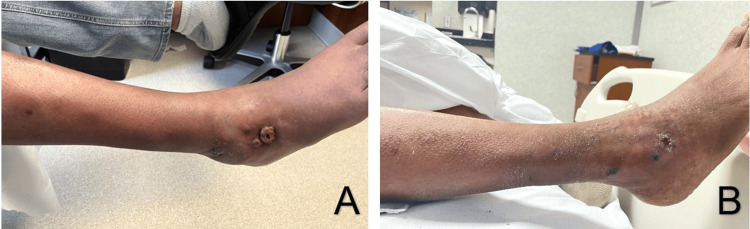
(A) Right lower extremity lesions pre-treatment. (B) Lesions at discharge.

While precise minimum inhibitory concentration (MIC) breakpoints have not been established for *Aspergillus ustus*, the antifungal sensitivities showed susceptibility to micafungin and posaconazole. Before receiving the sensitivity results, the patient was started on amphotericin B 3 mg/kg/day intravenous (IV) therapy and itraconazole 372 mg IV daily. Once the sensitivities were reported, the patient’s treatment was changed to amphotericin B 3 mg/kg/day IV and micafungin 200 mg/day IV. Over the next two months, the lesions largely resolved on this treatment regimen (Figure 4A, 4B). The patient was discharged with oral posaconazole 300 mg daily. He continued treatment until he died of a myocardial infarction six months after discharge.

## Discussion

When creating a differential diagnosis for a cutaneous infection that presents with a sporotrichoid pattern of spread, there are limited possible infectious etiologies. The most common causes include *Sporothrix schenckii*, *Nocardia brasiliensis*, *Mycobacterium marinum*, and *Leishmania brasiliensis* [6]. There have been very few reported cases of a sporotrichoid spread associated with *Aspergillus* spp. Of these case reports, none have identified *Aspergillus ustus* as the causative organism.

*Aspergillus ustus* rarely causes human disease and primarily affects immunocompromised individuals. It belongs to the *Aspergillus *section *Usti*, which has over 20 distinct but related species found worldwide in soil, caves, and indoor air [7,8]. A clinical review by Glampedakis et al. notes that *Aspergillus ustus *exhibits a high rate of therapeutic failure and reduced susceptibility to azoles [7].

When performing a literature search, only four other cases of cutaneous aspergillosis distributed in a sporotrichoid pattern have been reported. This demonstrates the rarity of cutaneous *Aspergillus *presenting with a sporotrichoid pattern of spread. These cases are summarized in Table 3.

**Table 3:** Summary of case reports of cutaneous aspergillosis infections with sporotrichoid spread M = male; CGD = chronic granulomatous disease; GMS = Gomori methenamine silver; PAS = periodic-acid-Schiff; IV = intravenous.

Article	Age/Sex	Immune Status	Presentation	Fungal Cause	Diagnostics	Treatment	Outcome
Palmero et al., 2009 [9]	3/M	CGD	Left forearm erythematous plaques, sporotrichoid spread with large axillary mass	Aspergillus fumigatus	Fungal culture of aspirate from left axillary lymph node	IV amphotericin B 1 mg/kg/day for two weeks, transitioned to oral voriconazole 100 mg twice daily for two months, then developed photosensitivity reaction, transitioned to oral itraconazole 70 mg twice daily	Concurrent pulmonary aspergillosis at diagnosis, later disseminated with splenic and mesenteric involvement
Osório et al., 2012 [10]	52/M	Etanercept 50 mg weekly for 3 years for treatment of plaque psoriasis and psoriatic arthritis	Right forearm lesions with sporotrichoid spread after a rabbit bite, and left periorbital lesion likely secondary to self-inoculation	Aspergillus fumigatus	Fungal culture on Sabouraud medium with gentamicin and chloramphenicol	Oral itraconazole 100 mg twice daily for four weeks	Complete resolution
								Verma et al., 2015 [11]	45/M	Immunocompetent	Right lower limb lesion with sporotrichoid spread, purulent discharge, and edema	Aspergillus nidulans	Combination of histopathology, GMS, and fungal culture	Oral itraconazole 200 mg twice daily (length unspecified to resolution), continued further for three months after	Complete resolution
																Pathania et al., 2022 [12]	29/M	Immunocompetent	Left upper limb papulonodular lesions with sporotrichoid spread and central ulceration	Aspergillus chivalieri	Combination of GMS, PAS, and fungal cultures, diagnostic confirmation with 18 rRNA sequencing	Oral itraconazole 200 mg twice daily for 12 weeks	Satisfactory resolution

The previous case reports of cutaneous aspergillosis with a sporotrichoid spread reveal a combination of both immunocompromised and immunocompetent patients (Table 3). In the cases of Verma et al. and Pathania et al., there were no identifiable causes of the infections related to immunosuppression, trauma, or environmental exposures [11,12]. In the case of Osório et al., the patient’s disease was directly associated with trauma, specifically a rabbit bite [10].

The diagnostic utility of several tests varied across the cases (Table 3). In the cases of Palmero et al. and Osório et al., microbiology identified the fungal element; however, histopathology and special stains were nondiagnostic or negative [9,10]. In the cases of Verma et al. and Pathania et al., both microbiology and pathology were essential to establish the diagnosis [11,12]. Oral itraconazole was successfully used for satisfactory or complete resolution in all cases, except for Palmero et al. This case, however, was complicated by newly diagnosed chronic granulomatous disease (CGD), and the choice to use itraconazole was influenced by an adverse drug reaction to voriconazole [9].

As seen in the present case and two of the previous case reports, histopathology did not aid in identifying the causative organism. Features that may lead to a lack of organism detection using histopathology include inadequate tissue sampling, low fungal burden, and previous treatment with antifungal therapies [5,13]. While histopathology is a valuable tool for the diagnosis of *Aspergillus *infections, diagnostic accuracy can be suboptimal and insensitive according to the 2016 guidelines from the Infectious Disease Society of America. They state that histopathologic examination should not solely be used for organism diagnosis due to false negatives and the possibility of atypical features [5]. This highlights the importance of fungal culture, as negative IHC and GMS stains alone cannot be used to rule out a fungal infection.

While the causative organism was unknown, the decision was made to treat the lesions with thermotherapy. Thermotherapy has shown efficacy in treating cutaneous fungal infections such as sporotrichosis, chromoblastomycosis, and other opportunistic mycoses, as well as cutaneous parasitic infections such as leishmaniasis. Thermotherapy can be given in conjunction with systemic therapy and is associated with minimal side effects. Thermotherapy may be a feasible alternative treatment for patients who cannot tolerate systemic therapy or for infections resistant to standard antimicrobial therapy [14]. Due to the safety and effectiveness of thermotherapy for treatment of several organisms on the initial differential diagnosis, the decision was made to treat the lesions with thermotherapy, pending identification of the causative organism.

Once the diagnosis of *Aspergillus ustus* was established, the patient could be started on systemic therapy. Treatment guidelines from the Infectious Disease Society of America, published in 2016, recommend voriconazole with possible surgical debridement in cases of burns or large soft tissue involvement. Alternative therapy includes amphotericin B, other azoles, and echinocandins [5]. A meta-analysis from Liu et al. (2024) reviewed 12 studies for optimizing the treatment of invasive aspergillosis. Isavuconazole was found to be the most effective agent and has the highest mortality benefit, followed by voriconazole and posaconazole. A combination of amphotericin B and caspofungin was also found to be highly effective [15].

*Aspergillus ustus* infections can be particularly challenging to treat. A retrospective study performed by Glampedakis et al. found that this species exhibits some level of azole resistance [7]. The antifungal sensitivities for this case demonstrated resistance to fluconazole and itraconazole, consistent with these findings. Currently, there are no validated MIC breakpoints for *Aspergillus ustus* to guide treatment. Optimal antifungal therapy remains unclear, emphasizing the key importance of utilizing antifungal sensitivities to guide treatment even in the absence of established breakpoints.

## Conclusions

Cutaneous *Aspergillus *infections are rare and may present with atypical patterns, making diagnosis challenging. This report presents a rare case of cutaneous *Aspergillus ustus* infection following trauma and environmental exposure, presenting with a sporotrichoid pattern of spread. Pathology is often critical in identifying the cause of atypical or nonspecific cutaneous infections. However, it should not be the only diagnostic technique utilized. Microbiology may reveal what pathology does not, as seen in this case. Thus, using a combination of both allows for the highest diagnostic accuracy. Furthermore, anti-infective drug sensitivities for fungi can provide critical information to rationally guide treatment. A comprehensive diagnostic approach is critical for identifying and treating uncommon cutaneous fungal infections.

## References

[1] Bolognia JL (2024). Dermatology - E-Book, 5th ed. Chantilly.

[2] Visagie CM, Houbraken J, Overy DP (2025). From chaos to tranquillity: A modern approach to the identification, nomenclature and phylogeny of Aspergillus, Penicillium and other Eurotiales, including an updated accepted species list. Stud Mycol.

[3] Quereshi S, Paralikar P, Pandit R, Razzaghi-Abyaneh M, Kon K, Rai M (2016). Pulmonary aspergillosis: Diagnosis and treatment. The Microbiology of Respiratory System Infections.

[4] Sugui JA, Kwon-Chung KJ, Juvvadi PR, Latgé JP, Steinbach WJ (2014). Aspergillus fumigatus and related species. Cold Spring Harb Perspect Med.

[5] Patterson TF, Thompson GR 3rd, Denning DW (2016). Practice guidelines for the diagnosis and management of aspergillosis: 2016 Update by the Infectious Diseases Society of America. Clin Infect Dis.

[6] Tobin EH, Jih WW (2001). Sporotrichoid lymphocutaneous infections: Etiology, diagnosis and therapy. Am Fam Physician.

[7] Glampedakis E, Cassaing S, Fekkar A (2021). Invasive aspergillosis due to Aspergillus section Usti: A multicenter retrospective study. Clin Infect Dis.

[8] Houbraken J, Due M, Varga J, Meijer M, Frisvad JC, Samson RA (2007). Polyphasic taxonomy of Aspergillus section Usti. Stud Mycol.

[9] Palmero ML, Pope E, Brophy J (2009). Sporotrichoid aspergillosis in an immunocompromised child: A case report and review of the literature. Pediatr Dermatol.

[10] Osório F, Magina S, Azevedo F (2012). Primary cutaneous aspergillosis complicating tumor necrosis factor-α blockade therapy in a patient with psoriasis. Actas Dermosifiliogr.

[11] Verma R, Vasudevan B, Sahni AK, Vijendran P, Neema S, Kharayat V (2015). First reported case of Aspergillus nidulans eumycetoma in a sporotrichoid distribution. Int J Dermatol.

[12] Pathania V, Sandhu S, Sengupta P, Kaur K (2022). Cutaneous aspergillosis masquerading in sporotrichoid morphology in an immunocompetent host. Med J Armed Forces India.

[13] Thompson GR 3rd, Le T, Chindamporn A (2021). Global guideline for the diagnosis and management of the endemic mycoses: An initiative of the European Confederation of Medical Mycology in cooperation with the International Society for Human and Animal Mycology. Lancet Infect Dis.

[14] Badgwell Doherty C, Doherty SD, Rosen T (2010). Thermotherapy in dermatologic infections. J Am Acad Dermatol.

[15] Liu A, Xiong L, Wang L, Zhuang H, Gan X, Zou M, Wang X (2024). Compare the efficacy of antifungal agents as primary therapy for invasive aspergillosis: A network meta-analysis. BMC Infect Dis.

